# Melorheostosis: a rare entity: a case report

**DOI:** 10.11604/pamj.2014.18.251.4661

**Published:** 2014-07-26

**Authors:** Abdelhakim Kherfani, Hachem Mahjoub

**Affiliations:** 1 A Orthopedics adults surgery department, MT Kassab Institute, La Manouba, Tunisia

**Keywords:** Melorheostosis, sclerotic bone dysplasias, CT, MRI

## Abstract

Melorheostosis is a rare entity belonging to the group of sclerotic bone dysplasias. Described for the first time in 1922 by Leri, it remains imperfectly known as clinical presentations are highly variable, and the etiological diagnosis is not fully elucidated. We report a case of polyostoticmelorheostosis for which radiological investigations were complete, in order to study this disease.

## Introduction

Melorheostosis is a rare benign non hereditary condition. It is known as a mesoderm sclerotic bone dysplasia, characterized by cortical hyperostosis with or without retraction of soft tissue. It was described for the first time in 1922 by Leri and Joanny as a dripping candle wax hyperostosis [1]. Since then, 300 cases have been reported in the literature. Its incidence is estimated 0.9 per million population [2]. It affects both men and women at any age. The locations are highly variable, and mono ostotic forms were more often described. Polyostotic forms localized to the lower limbs are rare. The disease can remain silent and be discovered incidentally. If not, symptoms are made of distortion, variable pain or limitation of joint mobility. It is a disease whose etiology is imperfectly understood, the genetic factors and metabolic predisposition or malformation of the vessels are proposed, but the exact cause is to be determined [3–5]. The diagnosis was facilitated by the recent imaging techniques, CT scan or better, the MRI that provides diagnostic certainty [6, 7]. Scintigraphy can detect subclinical lesions and monitor its progress [6, 7]. Therapeutic envisaged vary widely, depending on the discomfort and the localization.

## Patient and observation

**Figure 1:**
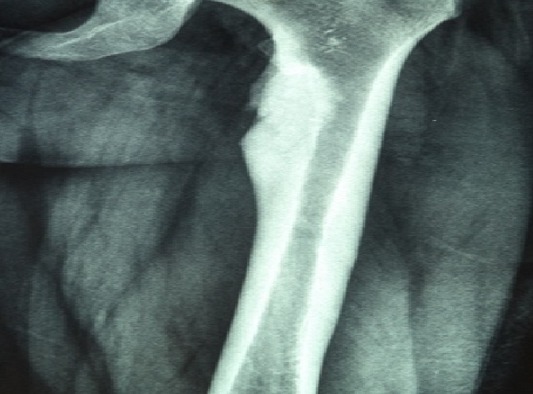
Flowing candle wax condensation in the proximal femur on a plane radiography, specific of the Leri's disease

**Figure 2:**
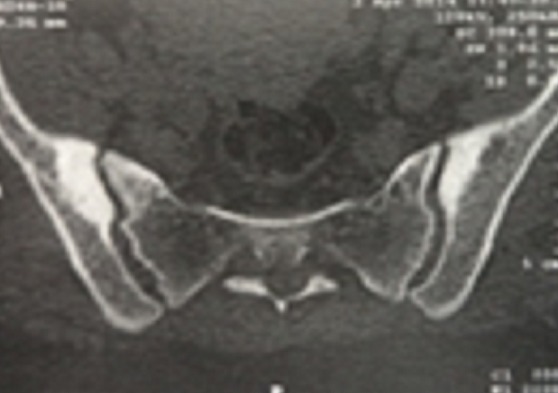
CT scan showing condensation of the iliac sides of sacro iliac joints

**Figure 3:**
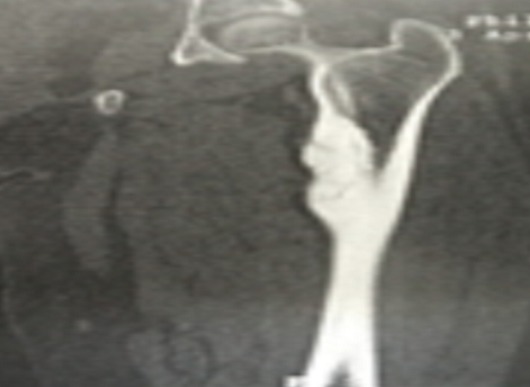
The caracteristic candle wax condensation of the proximal femur below the calcar in the CT scan

**Figure 4:**
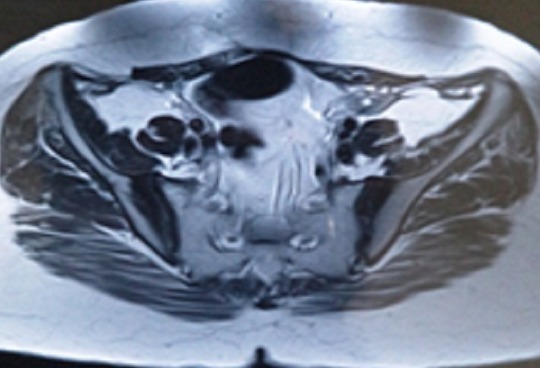
MRI on T2 sequence showing the condensation of the iliac side of the sacro iliac joints with no involvment of the joint nor the soft tissues

**Figure 5:**
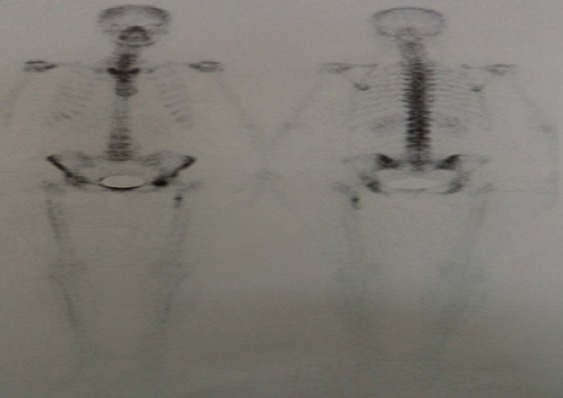
Scintigraphy showing hyper fixation of the proximal femur and the sacro iliac joints

We report the case of a 39 years-old female, with no past medical history, whose complaint was a spontaneous one week evolving isolated left cruralgia. The clinical examination was unremarkable apart from an anterior thigh pain at the mobilization of the left hip. No quadriceps hypotrophy, no fever and no limitations in the joints motion were found. Plain radiographs of the pelvis and proximal femur showed hypercondensation of the medial cortex of the left femur below the calcar, the classic flowing candle wax, combined with condensation of the iliac side of the sacroiliac joints bilaterally (Figure 1). Lab tests were normal. The diagnosis of melorheostosis was raised and we completed the explorations by a whole body bone scan that showed increased uptake in the proximal left femur and both sacroiliacs. CT of the pelvis and left femur was performed and showed an hypercondensation in the medial cortex of the left femur, and a bilateral condensation of the iliac side of the sacroiliac joints (Figure 2, Figure 3). The patient was put under analgesics and nonsteroidal anti -inflammatory drugs, with improvement of symptoms from J3 of hospitalization. An MRI was performed and showed the same bone condensation without extensions to the soft tissues (Figure 4). Given these findings, biopsy was not performed, and the patient was discharged to be followed in our consultation by standard radiographs every three months, and an annual bone scan. At last follow up, two years of the first consultation, two episodes of left cruralgia have been reported, and yielded by three days anti -inflammatory treatments. Scintigraphy (Figure 5) and plain radiographies remained similar to initial assessments.

## Discussion

Melorheostosis is still an imperfectly known pathology and many questions remain unanswered. The role of a genetic predisposition has been suggested, but evidence does not yet exist considering that familial cases have not yet been reported. Its association with vascular sector malformations and some observations reported changes in skin pigmentation suggests that a mutation may be responsible [3–5].

In this rare disease, the clinical presentations are highly variable and locations are diverse. Both upper or lower limbs can be affected in addition to the axial skeleton. The lower limbs are affected more often than the upper limbs. Conventional imaging is very characteristic when it shows the flowing candle wax, but this aspect can be seen in other conditions such as osteomyelitis or bone tumor. In some presentations it may be lawful to biopsy and perform bacteriological examinations [7]. In the most common cases, imaging may be sufficient to make the diagnosis. Cross-sectional imaging is very essential: CT shows bone lesions with its classical aspects and quantifies it in the spatial planes [6, 7] whereas MRI can describe the extension to the soft tissues and rule out possible differential diagnoses [6, 7]. Bone scintigraphy has an important contribution in melorheostosis since it can detect subclinical lesions and monitor progress. It also allows to differentiate the developmental stages according to the intensity of the bone fixation [6, 7]. We recommend monitoring by full body scans to detect any changes, especially since the evolution is unpredictable.

The therapeutic is not well codified. It is symptomatic in most cases, in order to control bone pain. Protocols based on Pamidronate [8] have been proposed and appear to improve symptoms but do not slow the progress. Surgery is indicated in cases where there is major axial deformation or consequent limitations of joint mobility. It is not easy and a knowledge of soft tissues involvement is a prerequisite.

## Conclusion

Melorheostosis is a rare, benign with chronic course, often with outbreaks interspersed with remissions. We emphasize the need to ensure the diagnosis by plane radiographies, CT scan or MRI. If any doubt, biopsy may be necessary. Scintigraphy is the best exploration for monitoring the disease. Treatment is symptomatic essentially, based on nonsteroidal anti-inflammatory. Surgery can have its place in large deformations and joint damage, but with great care.
